# Cell-type-specific regulation of acute respiratory distress syndrome by P2RY_6_-expressing CD11c^+^ cells

**DOI:** 10.3389/fimmu.2026.1940896

**Published:** 2026-10-26

**Authors:** Zahra Kargarpour, Bertram Aschenbrenner, Markus Kramer, Seda Metekol, Amra Begunic, Silvana Geleff, Eva Obermayr, Robert Zeillinger, Anastasia Papaporfyriou, Andreas Renner, Beate Lichtenberger, Daniela Gompelmann, Marco Idzko, Ahmed El-Gazzar

**Affiliations:** 1 Division of Pulmonology, Department of Internal Medicine II, Comprehensive Center for Chest Diseases, Medical University of Vienna, Vienna, Austria; 2 Department of Dermatology, Medical University of Vienna, Vienna, Austria; 3 Department of Pathology, Medical University of Vienna, Vienna, Austria; 4 Molecular Oncology Group, Department of Obstetrics and Gynecology, Comprehensive Cancer Center-Gynecologic Cancer Unit, Medical University of Vienna, Vienna, Austria; 5 Department of Pneumology, Medical Center, University of Freiburg, Freiburg, Germany

**Keywords:** acute respiratory distress syndrome, CD11c-expressing cells, LPS-induced lung injury, neutrophils, P2RY_6_, macrophages

## Abstract

Acute respiratory distress syndrome (ARDS) is a life-threatening inflammatory lung disorder characterized by excessive neutrophilic inflammation and increased microvascular permeability. We previously demonstrated the involvement of the G_q_-coupled P2Y_2_ purinergic receptor (P2RY_2_) in ARDS pathogenesis. Here, we investigated the contribution of the related purinergic receptor P2Y_6_ (P2RY_6_) and defined its cell-type-specific functions using a preclinical mouse model of ARDS. Wild-type female mice were treated with the selective P2RY_6_ antagonist MRS2578 through intratracheal administration using prophylactic and therapeutic approaches, followed by lipopolysaccharide (LPS) challenge. The role of P2RY_6_ was further evaluated in global *P2ry_6_*-deficient mice and conditional knockout models targeting specific cellular compartments. Bronchoalveolar lavage (BAL) samples were analyzed by flow cytometry and multiplex cytokine assays, and lung tissues were assessed by histological and immunohistochemical analyses. Pharmacological inhibition of P2RY_6_ significantly reduced Ly6G^+^F4/80⁻ neutrophil accumulation, vascular leakage and pro-inflammatory cytokine and chemokine production in the ARDS model. Consistently, *P2ry_6_*-deficient mice exhibited reduced Ly6G^+^F4/80⁻ neutrophil recruitment and attenuated inflammatory responses following LPS challenge. Cell-type-specific deletion revealed that P2RY_6_ deficiency in Vav-expressing hematopoietic cells and CD11c-expressing cells reproduced the protective phenotype observed in global *P2ry_6_* knockout mice. In contrast, P2RY_6_ deletion in CD4^+^ T cells, *Scgb1a1*-expressing airway epithelial cells or LysM-expressing myeloid cells did not significantly alter ARDS-associated inflammation. Collectively, our findings identify CD11c-expressing P2RY_6_^+^ cells as critical regulators of ARDS pathogenesis and highlight P2RY_6_ as a potential therapeutic target for controlling inflammatory lung injury.

## Graphical Abstract

**Figure:**
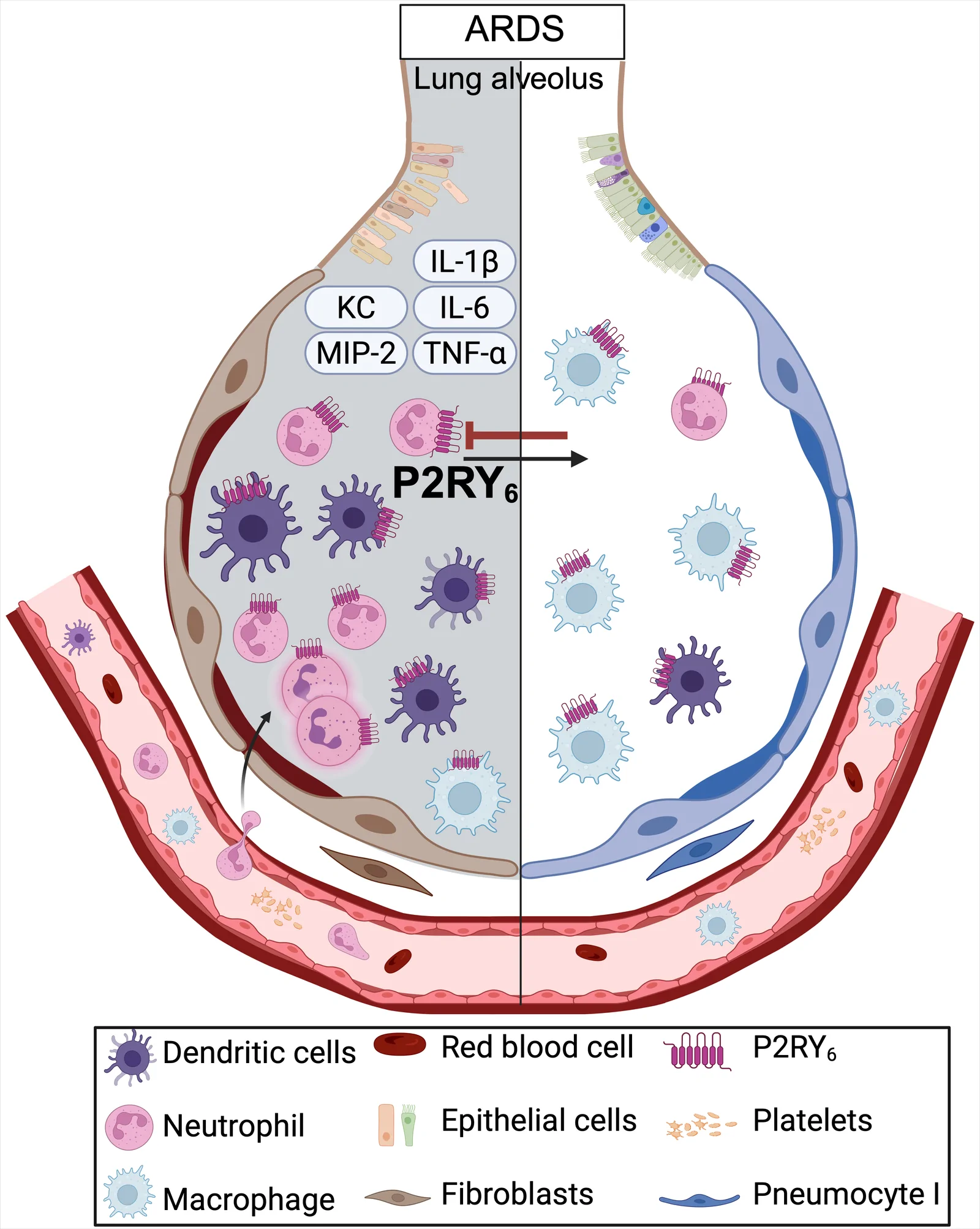
Schematic summary of the cell-type-specific role of P2RY_6_-expressing CD11c⁺ cells in ARDS pathogenesis.

## 1 Introduction

Acute respiratory distress syndrome (ARDS) is a life-threatening condition commonly associated with lung infections and systemic inflammation. It is characterized by rapid onset and progression (1, 2). The pathological progression of ARDS can be divided into three overlapping phases: acute, subacute (proliferative), and chronic (fibrotic), representing a continuum from tissue injury to repair. The acute phase is characterized by diffuse alveolar damage, protein-rich pulmonary edema, and neutrophil infiltration resulting from endothelial and epithelial injury. Vascular injury represents a critical initiating event, promoting neutrophil recruitment through sequential endothelial interactions and subsequent migration into the lung tissue. During the proliferative phase, extracellular matrix deposition and fibrocellular plug formation become prominent, whereas the fibrotic phase is characterized by alveolar wall thickening, architectural distortion, cystic airspaces, and honeycombing associated with persistent microvascular injury (3, 4). Hallmark features of ARDS, including neutrophil accumulation, pulmonary edema, and severe hypoxemia, contribute to disease severity and increased mortality risk (5–7).

Despite advances in ventilatory strategies, critical care management, and imaging technologies over the past two decades, effective pharmacological therapies for ARDS remain unavailable (8–11). The limited success of clinical trials highlights the need to better understand ARDS pathogenesis and identify novel therapeutic targets (2). ARDS is characterized by a dysregulated inflammatory response that drives lung injury and disease progression (12). Bacterial pneumonia and sepsis are among the most common triggers of ARDS. Neutrophils play a central role in this process by promoting endothelial barrier disruption and immune-mediated pulmonary tissue damage (3, 13). Following injury, neutrophils are rapidly recruited to the lung, and excessive neutrophil activation and accumulation at sites of injury represent key features of acute inflammation (14–16).

During inflammatory processes or hypoxic conditions, cellular stress responses promote the release of extracellular nucleotides, including adenosine-5′-triphosphate (ATP), adenosine-5′-diphosphate (ADP), uridine-5′-triphosphate (UTP), and uridine-5′-diphosphate (UDP) (17–19). These extracellular nucleotides act as signaling molecules through activation of P2 purinergic receptors (P2Rs), regulating diverse cellular responses (20). P2Rs comprise two major families: G protein-coupled P2Y receptors, consisting of nine subtypes (P2Y_1_, P2Y_2_, P2Y_4_, P2Y_6_, P2Y_8_, P2Y_11_, P2Y_12_, P2Y_13_, and P2Y_14_), and ligand-gated ion channel P2X receptors, consisting of seven subunits (P2X_1_–P2X_7_) (18, 21). Activation of P2Rs regulates multiple biological processes, including immune cell migration, cytokine secretion, reactive oxygen species production, and apoptosis. Functional P2Rs are expressed by both inflammatory and structural cells in the lung (18, 22). Several P2R subtypes have been implicated in pulmonary diseases, including ARDS, bronchial asthma and chronic obstructive pulmonary disease (22–24). In respiratory inflammation, purinergic signaling regulates immune cell functions, including neutrophil chemotaxis, activation and survival during bacterial lung infection and ARDS (25, 26). Neutrophils and macrophages express functional P2Y and P2X receptors, whose activation regulates inflammatory responses, including pro-inflammatory cytokine production (26, 27). Although P2X_7_ and P2Y_2_ have been implicated in lung injury and LPS-induced ARDS (26, 28), the contribution of other P2 receptor subtypes to ARDS pathogenesis remains poorly understood.

In this study, we provide evidence that P2RY_6_ contributes to acute LPS-induced ARDS in female mice. Pharmacological inhibition of P2RY_6_ reduced Ly6G^+^F4/80⁻ neutrophil accumulation, plasma leakage and pro-inflammatory cytokine and chemokine production. Consistently, global *P2ry6*-deficient mice exhibited attenuated neutrophil recruitment and inflammatory responses following LPS challenge. To determine whether these effects were cell-type specific, we examined conditional *P2ry6* deletion in distinct cellular compartments. Notably, deletion of *P2ry6* in CD11c-expressing cells reproduced the protective phenotype observed in globally deficient mice, whereas deletion in other targeted cell populations did not significantly alter ARDS-associated inflammation. Furthermore, P2RY_6_ expression was increased in lung tissue and immune cells following LPS challenge. Together, these findings identify CD11c-expressing P2RY_6_^+^ cells as important regulators of LPS-induced lung inflammation and suggest that targeting P2RY_6_ signaling may represent a potential therapeutic strategy for ARDS, particularly in females.

## 2 Materials and methods

### 2.1 Mice

Female C57BL/6 mice (6-10-week-old), including wild type, *P2ry_6_^fl/fl^*, *Vav*-Cre × *P2ry_6_^fl/fl^*, *CD11c*-Cre × *P2ry_6_^fl/fl^*, *LysM*-Cre × *P2ry_6_^fl/fl^*, *CD4*-Cre × *P2ry_6_^fl/fl^*, *CCT*-Cre × *P2ry_6_^fl/fl^* mice, were used in all experiments. All mice were bred and housed in the animal facilities of the Medical University of Vienna and the University Hospital Freiburg. The animal experiments described within this study were approved by the Animal Ethics Committee of the Medical University of Vienna (Austria) and the Animal Ethics Committee of the University of Freiburg (Germany). All mice were maintained in specific pathogen-free (SPF) conditions. Genetically manipulated mice used in this study were described previously (29). To stimulate an LPS-induced acute lung inflammation, mice were first anesthetized using intraperitoneal (i.p.) injection of ketamine (100 mg/kg body weight, VetViva Richter GmbH, Austria) and xylazine (5 mg/kg body weight, Elanco, IN, USA) and then were intratracheal (i.t.) instilled with LPS (1000 μg/kg body weight, Santa Cruz, TX, USA, #sc-3535) from *E. coli* dissolved in phosphate-buffered saline (PBS) (Thermo Fisher Scientific, MA, USA, #14040091) to a total volume of 50 µL or with sterile PBS for control. The applied dosage was based on previous studies (29, 30). Mice were euthanized 24 hours after LPS or PBS administration by i.p. injection of five-fold overdoses of ketamine (500 mg/kg body weight, VetViva Richter GmbH, Austria) and xylazine (25 mg/kg body weight, Elanco, IN, USA) depending on the experiment setting. In the therapeutic treatment experiments, mice were euthanized 24 hours after MRS2578 or PBS administration. For sorting of lung epithelial cells, lung tissue was minced and collected in RPMI medium (Gibco, USA, CA, #11875093) for RNA extraction experiment.

### 2.2 Purinergic receptor antagonist application

The P2RY_6_ selective inhibitor MRS2578 (TOCRIS Bioscience, UK, #2146) was administered i.t. (10 µM in 50 µL total volume) 30 minutes prior (prophylactic, n= 5) or 24 hours after (therapeutic, n= 5) i.t. LPS instillation. At the endpoint of LPS/inhibitor challenge, mice were euthanized and a BAL sample was obtained by cannulation of the trachea. Lungs were perfused by injecting of 3 mL PBS. The remaining lung tissue was slowly filled with histofix and transferred in formalin solution for histology analysis. The number of neutrophils and macrophages in the BAL was quantified by differential cell counting of BAL cytospin preparations and flow cytometry.

The mice received an i.t. administration of either vehicle (PBS, n= 3-5), or 10 µM MRS2578, in 50 µL total volume 30 minutes prior (prophylactic, n= 5) or 24 hours after LPS instillation (therapeutic, n=5). The concentrations were chosen based on established literature (31) and preliminary dose-response studies aiming to achieve effective receptor blockade within our experimental system. Mice were euthanized 24 hours after both LPS application in the prophylactic and antagonist treatment in the therapeutic setting.

### 2.3 Collection of lung tissue, bronchoalveolar lavage fluid and cells

The animals were euthanized by i.p. injection of five-fold overdoses of ketamine (500 mg/kg body weight) and xylazine (25 mg/kg body weight) and BAL cells were collected. Briefly, the lungs were first flushed once with 0.9 mL of PBS. Then, they were flushed thrice more with an additional 0.7 mL each time. The first part was used for multiplex assays to determine cytokine and chemokine concentrations, while cells from all washes were pooled for flow cytometry. The BAL fluid (BALF) was kept on ice until processing.

### 2.4 Total cell count and flow cytometry analysis

The BAL was centrifuged (7 minutes, 330 g, 4 °C), and protease inhibitor 100x (Thermo Scientific, Germany, #1861279) was added to the supernatant of the first 0.9 mL recovered supernatants and stored at -80 °C for subsequent analysis of cytokine levels using multiplex assay. Red cells were lysed using red cells lysis buffer (0.2% NaCl followed by 1.6% NaCl, pH=7.4) and the cell pellets were resuspended in 100 µL PBS for total cell counting by hemacytometer. The differential cell count analysis was done by flow cytometry (CytoFLEX, BECKMAN COULTER; Krefeld, Germany) as previously described (23). Briefly, mouse BAL cells were stained with DAPI (Thermo Scientific, Germany, #D1306, 10 µg/test) or Zombie Yellow (Violet 610) (BioLegend, San Diego, CA, USA; #423113, 1:100) Live/Dead dye for 10 minutes. Cells were incubated with unlabeled rat anti-mouse CD16/CD32 antibody (Mouse BD FcR Blocking reagent, Miltenyi Biotec, #130092575, 3: 50) to block Fc receptors and stained for 10 minutes. The cells were then stained with anti-mouse Ly6G-FITC monoclonal antibody (clone 1A8; Miltenyi Biotec, #130-102–934 and #130-102-296, 1:50) and anti-mouse F4/80-PE-Vio^®^ 615 monoclonal antibody (Miltenyi Biotec, #130-123-913, 1:50) in PBS for 20 minutes at 4 °C in the dark. Following staining, cells were washed once with 1xPBS before flow cytometric analysis. After resuspension in 1xPBS, samples were immediately analyzed by flow cytometry. Isotype control antibodies were used to establish the gating strategy (**Supplementary Figure 1**). Ly6G^+^ or F4/80^+^ cells were reported as estimated numbers per mouse. Cell numbers were calculated from the number of gated events detected by flow cytometry, normalized to the recovered BAL fluid volume to account for differences in lavage recovery between samples, and scaled to the total lavage volume of 3 mL PBS instilled per mouse. For fluorescence-activated cell sorting (FACS) of BAL and lung cells, BAL cells were sorted using a BD FACS Aria I cell sorter available through the Unit Flow Cytometry of the Core Facilities of the Medical University of Vienna. The total number of BAL cells was 2.25 ± 0.79 × 10^6^ cells in the LPS group and 0.29 ± 0.20 × 10^6^ cells in the PBS group. For lung tissue analysis, lungs were resected for qPCR analysis and stored in Roswell Park Memorial Institute (RPMI) medium. Single-cell suspensions were prepared from murine lung tissues preserved in RPMI, as previously described (32), using a Single Cell Suspension Kit (Miltenyi Biotec, #130-110-201) according to the manufacturer’s instructions. The resulting cell suspensions were filtered and centrifuged, followed by red blood cell lysis. Cells were subsequently quantified and used for FACS sorting experiments. CD45-positive leukocytes, neutrophils, macrophages, CD11c-positive cells and CD4^+^ T cells were identified and isolated from BAL cells in independent sorting experiments using anti-CD45 PerCP (#130-110-636, 1:50), anti-Ly-6G (clone 1A8) FITC-conjugated, anti-F4/80 PE-Vio 615-conjugated, anti-CD11c VioBlue (#130-122-939, 1:50) and anti-CD4 FITC (#130-102-541, 1:50) antibodies, respectively. EpCAM^+^ epithelial cells were isolated from lung tissue in a separate sorting experiment using anti-EpCAM PE-Vio 770 (#130-102-165, 1:50) antibody. All antibodies were purchased from Miltenyi Biotec. The gating strategy was established based on the isotope control sample (**Supplementary Figure 2**).

### 2.5 Cytokine and chemokine assays

The levels of 17 cytokines and chemokines in mouse BALF were measured using the Bio-Plex Pro Mouse Th17 Assays (Bio-Rad Laboratories, #12010828), following the manufacturer’s instructions. This assay is a multiplex bead-based system that works similarly to a sandwich enzyme-linked immunosorbent assay (ELISA), allowing multiple analytes to be measured at the same time. The assay uses color-coded beads, each specific to a different analyte. These beads are first identified by a red laser, and the amount of bound analyte is then detected by measuring the fluorescence of a reporter molecule (phycoerythrin, PE) using a green laser. BAL samples and standards were prepared in 1xPBS containing 0.5% BSA to reduce nonspecific binding. All samples and standards were measured in duplicate. Standard curves were generated using a five-parameter logistic (5-PL) model and analyte concentrations were calculated based on the median fluorescence signal from at least 50 beads per analyte, using an eight-point standard curve. Respective measurements were performed using the Bio-Plex 200 instrument (Bio-Rad Laboratories). For evaluation of cytokines and chemokines obtained from inhibitor-treated mice in **Figures 1**, **2** an R&D Systems assay was applied according to the manufacturer’s instructions. The different assay platforms have distinct calibration systems and quantitative ranges.

**Figure 1:**
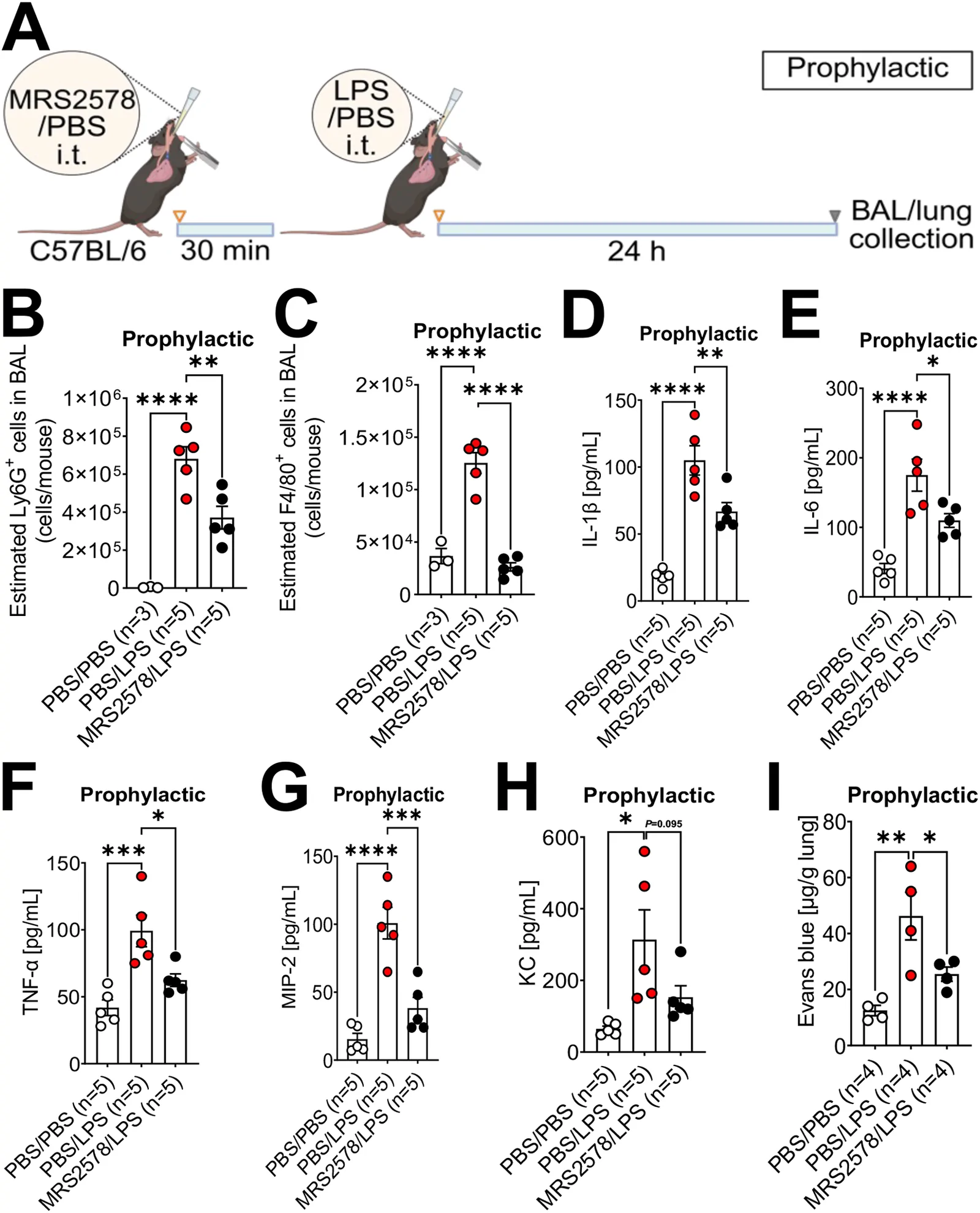
Pharmacological inhibition of P2RY_6_ attenuates inflammatory features of LPS-induced ARDS during prophylactic treatment. **(A)** Schematic representation of the prophylactic treatment protocol. Wild-type female mice received i.t. administration of MRS2578 or PBS, followed 30 minutes later by i.t. administration of LPS or PBS. Mice were euthanized 24 hours after LPS challenge. **(B, C)** Estimated numbers of Ly6G^+^F4/80⁻ neutrophils and F4/80^+^Ly6G⁻ macrophages in BAL fluid, expressed as cells per mouse, were quantified by flow cytometry following prophylactic P2RY_6_ inhibition. Data are presented as estimated cell numbers per mouse. n indicates the number of mice analyzed. **(D–H)** Concentrations of pro-inflammatory cytokines (IL-1β, IL-6, and TNF-α) and chemokines (MIP-2 and KC) in BAL were measured following prophylactic treatment using an R&D Systems. **(I)** Pulmonary vascular leakage was assessed by Evans blue–albumin extravasation. Evans blue dye (20 mg/kg) was intravenously injected via the tail vein and dye accumulation in lung tissue was quantified spectrophotometrically. Data are presented as mean ± SEM. Statistical analysis was performed using ordinary one-way ANOVA followed by Šídák’s multiple-comparisons test, comparing each treatment group with the LPS control group (PBS/LPS). *P < 0.05, **P < 0.01, ***P < 0.001, ****P < 0.0001.

**Figure 2:**
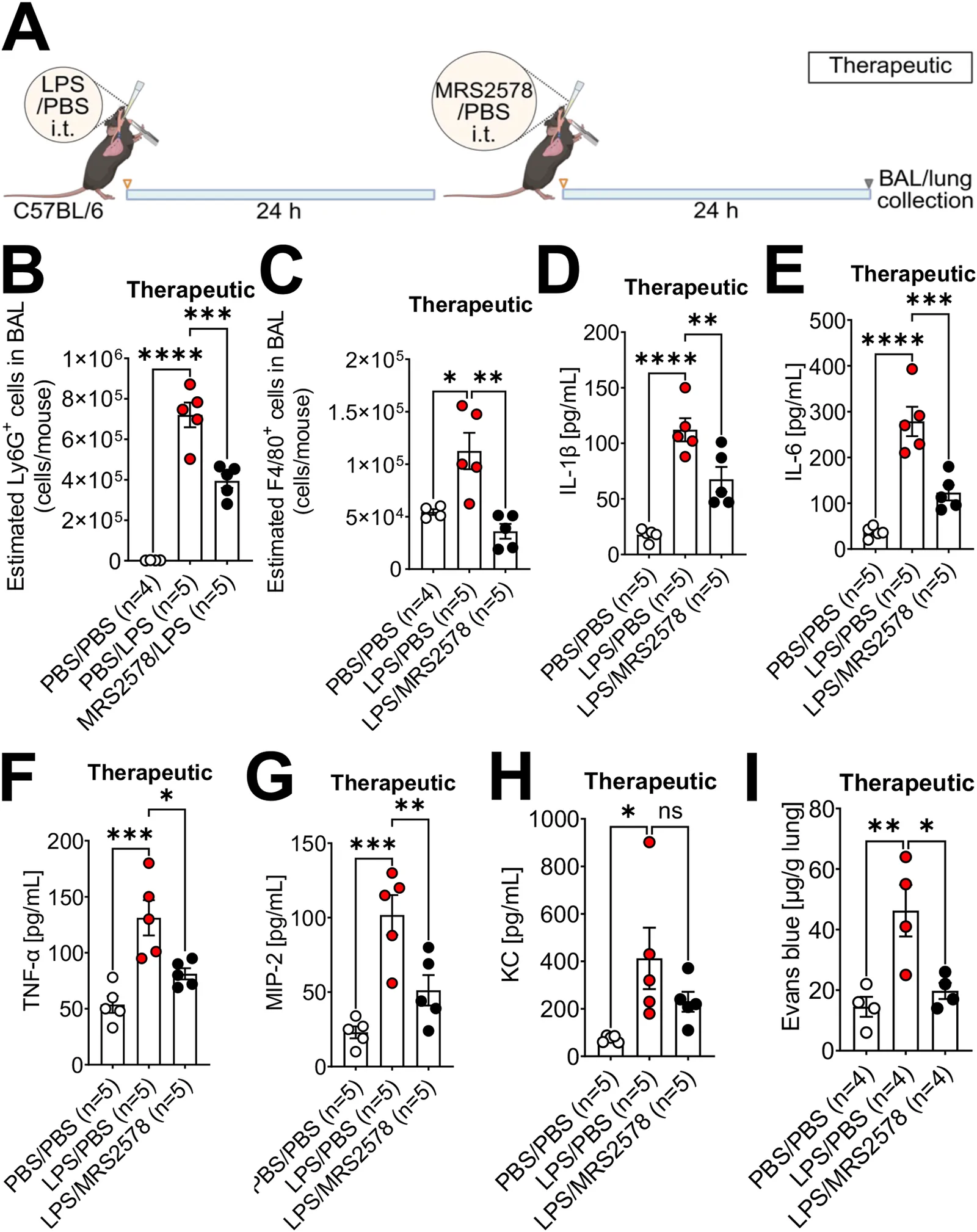
Pharmacological inhibition of P2RY_6_ attenuates inflammatory features of LPS-induced ARDS during therapeutic treatment. Wild-type female mice received i.t. administration of LPS or PBS, followed 24 hours later by i.t. administration of MRS2578 or PBS. Mice were euthanized 24 hours after P2RY_6_ inhibitor administration. **(A)** Schematic representation of the therapeutic treatment protocol in the ARDS mouse model. **(B, C)** Estimated numbers of Ly6G^+^F4/80⁻ neutrophils and F4/80^+^Ly6G⁻ macrophages in BAL fluid, expressed as estimated number of cells per mouse, were quantified by flow cytometry following therapeutic P2RY_6_ inhibition. n indicates the number of mice analyzed. **(D–H)** Concentrations of pro-inflammatory cytokines (IL-1β, IL-6 and TNF-α) and chemokines (MIP-2 and KC) in BAL were measured following therapeutic treatment using an R&D Systems **(I)** Pulmonary vascular leakage was assessed by Evans blue–albumin extravasation. Evans blue dye (20 mg/kg) was intravenously injected via the tail vein, and dye accumulation in lung tissue was quantified spectrophotometrically. Data are presented as mean ± SEM. Statistical analysis was performed using ordinary one-way ANOVA followed by Šídák’s multiple-comparisons test, comparing each treatment group with the LPS control group (PBS/LPS). *P < 0.05, **P < 0.01, ***P < 0.001, ****P < 0.0001. ns, not significant.

### 2.6 cDNA Synthesis and qRT-PCR

To obtain the cDNA from different sorted cells, we used the SuperScript™ IV single cell/low input cDNA PreAmp Kit (Thermo-Fisher Scientific GmbH, Germany, #11752048) according to manufacturer’s instructions. Briefly, up to 1000 cells per sample were used to perform the lysis reaction and immediately proceed to conduct the RT reaction based on the protocol. Subsequently, the preamplification step was done and the cDNA stored at -20 °C. Quantitative real-time PCR (qRT-PCR) was performed using Luna Universal qPCR Master Mix (NEB, #M3003E) and FAM-labelled TaqMan probes. Fluorescence was detected using the 483–533 nm channel. Gene expression was quantified using the 2^−ΔΔCt^ method, with *Gapdh* used as the reference gene for normalization. The results are presented as relative expression (2^−ΔΔCt^) with the following murine gene-specific Taqman probes: *P2ry_6_* (Mm02620937) and control *Gapdh* (Mm99999915_g1), both from Applied Biosystems. PCR amplification was performed using the LightCycler 480 Instrument II (LC480) Real-Time PCR System (Roche) under the following cycling conditions: enzyme activation at 95 °C for 5 minutes and 45 amplification cycles consisting of denaturation at 95 °C for 15 seconds and annealing/extension at 60 °C for 1 minute. The results are analyzed using 2^-ΔΔCt^ values normalized to *Gapdh.*

### 2.7 Plasma leakage assay

To assess alterations in alveolar–capillary membrane permeability, pulmonary vascular leakage was evaluated using the Evans blue–albumin extravasation assay as previously described (33). Briefly, Evans blue dye conjugated to albumin (EBA, Sigma-Aldrich, Germany, #314136; 20 mg/kg) was administered intravenously via the tail vein (n= 4). After 30 minutes, mice were euthanized, and lungs were perfused with PBS containing 5 mM EDTA to remove intravascular dye. The perfused lungs were excised en bloc, dried, weighed and snap-frozen in liquid nitrogen. Lung tissue was homogenized in PBS (1 mL per 100 mg tissue) and incubated in formamide at 60 °C for 18 hours to extract Evans blue dye. Following centrifugation at 5, 000 × g for 30 minutes, the optical density of the supernatant was measured spectrophotometrically at 620 nm. Evans blue extravasation was quantified using a standard curve and expressed as µg of Evans blue dye per gram of lung tissue (33).

### 2.8 Polymorphonuclear neutrophil migration into the BAL and interstitium

Evaluation of polymorphonuclear neutrophil (PMN) migration was performed as previously described (26, 34). Briefly, at different time points after LPS exposure alone or together with MRS2578, the mice were injected intravenously with Alexa 633-labeled rat anti-mouse GR-1 (10 µg, #A-21126) antibody and allowed to circulate for 5 minutes to bind intravascular PMN and then they were killed (n=3-4). After performing BAL, total lung tissue was harvested. BAL was determined by flow cytometry and the lungs were minced. Then, the lungs were digested with 125 U/mL collagenase type XI (Sigma Aldrich, Germany, #C7657), 60 U/mL hyaluronidase type I-s (Sigma Aldrich, MO, USA #H3506), and 60 U/mL DNase (Sigma Aldrich, MO, USA, #AMPD1) at 37 °C for 30 minutes. The digested lungs were obtained by being passed through a cell strainer and the cell suspension was centrifuged for 10 minutes at 300 g. The pellet was lysed with red blood-lysis buffer (ammonium chloride lysis buffer, 150 mM NH_4_Cl, 10 mM KHCO_3_, 100 nM EDTA, dH_2_O, pH=7.4) to remove erythrocytes and centrifuged again. These recovered cells were counted and determined by flow cytometry (34).

### 2.9 Histology

Mice were euthanized, and lungs were resected and perfused with 4% formaldehyde (Carl Roth GmbH, Karlsruhe, Germany) before preparation of formalin-fixed paraffin-embedded (FFPE) sections and subsequent H&E (haematoxylin and eosin) staining. FFPE sections were deparaffinized by heating and incubation in Neo-Clear, followed by additional fresh Neo-Clear washes. After rehydration, sections were stained with filtered Mayer’s hematoxylin (Sigma-Aldrich; MHS-16), followed by rinsing in cool running double-distilled water. Sections were then differentiated in 0.1% HCl solution and counterstained with eosin. After rinsing, slides were dehydrated through graded ethanol solutions (95% and 100% EtOH), cleared in butyl acetate, and mounted with eukitt before coverslipping. All H&E staining and image analyses were performed by investigators blinded to the mouse group assignments. H&E-stained slides were scanned using a Vectra Polaris automated quantitative pathology imaging system (Akoya Biosciences, Marlborough, MA, USA). H&E-stained whole-slide images were visualized and manually assessed using the HALO^®^ platform (v3.6.4134; Indica Labs, Albuquerque, NM, USA). Neutrophil-rich zones were imaged at high magnification for detailed structural characterization.

### 2.10 SFOG staining

Saponin Fuchsin Orange G (SFOG) staining was performed to assess tissue morphology and extracellular matrix deposition. Tissue sections were deparaffinized in Bouin for two hours at 60 °C, rehydrated through a 70% ethanol and rinsed in distilled water. Sections were stained according to the SFOG protocol, which differentially stains fibrin, collagen and cytoplasmic components. Briefly, sections were stained with Weigert’s iron hematoxylin for nuclear visualization, rinsed in distilled water and incubated with 1% phosphomolybdic acid, followed by treatment with the SFOG staining solution containing acid fuchsin, Orange G and aniline blue. After washing, sections were mounted with a permanent mounting medium. SFOG-stained slides were scanned using a Vectra Polaris automated quantitative pathology imaging system (Akoya Biosciences, Marlborough, MA, USA). The resulting whole-slide images were visualized and manually assessed using the HALO^®^ platform (v3.6.4134; Indica Labs, Albuquerque, NM, USA).

### 2.11 Quantification of lung injury and erythrocyte tissue coverage

Quantification of lung injury and erythrocyte tissue coverage was performed in H&E- and SFOG-stained lung sections, partly using the HALO^®^ image analysis platform. For each animal, lung injury was assessed in 20 randomly selected microscopic fields distributed across all lung lobes at high magnification (400×). In each field, five key histopathological features of ARDS, as described in the Matute-Bello et al. paper (35), were evaluated using a three-point ordinal scoring system (0, 1, or 2): (A) neutrophils in the alveolar space, (B) neutrophils in the interstitial space, (C) hyaline membranes, (D) proteinaceous debris filling the airspaces, and (E) alveolar septal thickening. Following assessment of all 20 fields, the scores for the five histopathological features were combined according to the following formula: 20 × A + 14 × B + 7 × C + 7 × D + 2 × E − A − E, where A–E correspond to the five histopathological features described above. The resulting composite score was normalized by dividing by 20 × 100, where 20 represents the number of microscopic fields evaluated per animal, yielding a final histological lung injury score ranging from 0 to 1. Erythrocyte tissue coverage was assessed in SFOG-stained sections by quantifying the area occupied by erythrocytes within the lung tissue.

### 2.12 Statistical analysis

Data are expressed as mean ± SEM. Differences between two independent treatment groups were assessed using an unpaired Welch’s t-test, without assuming equal variances. Comparisons among three or more groups were analyzed using one-way analysis of variance (ANOVA), followed by Šídák’s or Tukey’s multiple-comparisons test, as appropriate and as indicated in the corresponding figure legends. Where applicable, data involving two independent variables were analyzed using two-way ANOVA, followed by Bonferroni’s multiple-comparisons test. For statistical analysis we used GraphPad Prism version 9 (GraphPad Software, USA). Differences were considered significant at P < 0.05 and highly significant at P < 0.005.

## 3 Results

### 3.1 Upregulation of *P2ry_6_* expression in lung tissue and distinct pulmonary cell populations following LPS-induced ARDS

Our previous study demonstrated that *P2ry_6_* mRNA expression is significantly upregulated in the lungs of mice with LPS-induced ARDS (26). Building on these findings, we investigated the expression profile of *P2ry_6_* across distinct cell populations isolated from BAL of WT mice 24 hours after intratracheal (i.t.) administration of LPS (n= 4-8) or PBS (n= 3-6). BAL cells were isolated and sorted into CD45-positive leukocytes, CD11c-expressing cells, Ly6G^+^ neutrophils, F4/80^+^ macrophages, and CD4^+^ T cells in independent sorting experiments. The average number of BAL cells used for sorting was 2.25 ± 0.79 × 10^6^ cells in the LPS group and 0.29 ± 0.20 × 10^6^ cells in the PBS group. EpCAM^+^ epithelial cells were isolated and sorted from a single-cell suspension derived from lung tissue. For low-abundance sorted populations, cDNA was generated from fewer than 1, 000 cells using a cDNA synthesis kit optimized for low-input samples, enabling subsequent qPCR analysis. LPS challenge significantly increased *P2ry_6_* mRNA expression in CD45-positive leukocytes, CD11c-expressing cells, Ly6G^+^ neutrophils, F4/80^+^ macrophages and CD4^+^ T cells, whereas no significant change was observed in epithelial cells (**Figures 3A–F**). These findings indicate that *P2ry_6_* induction occurs primarily in immune cell populations during LPS-induced lung inflammation. The gating strategy was established as shown (**Supplementary Figure 2**).

**Figure 3:**
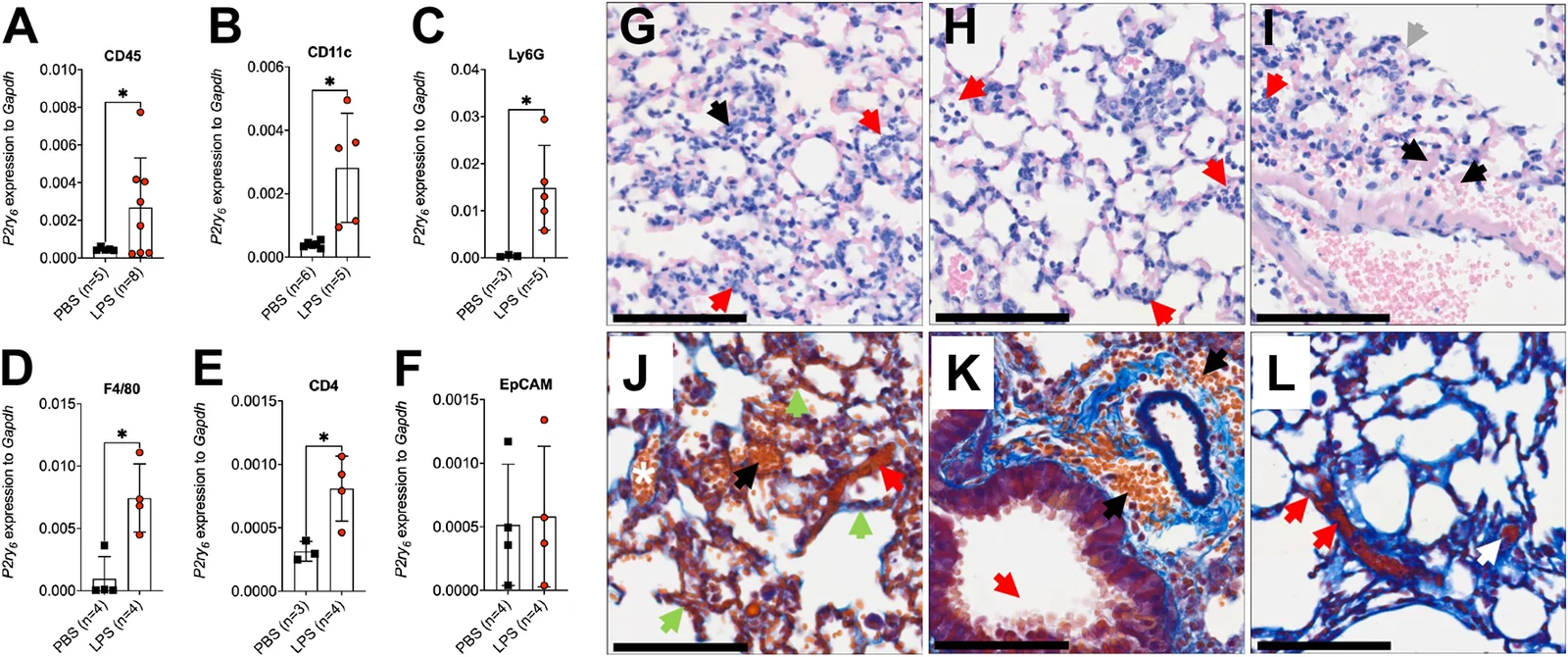
LPS-induced inflammation increases *P2ry_6_* expression in immune cell populations and induces characteristic lung inflammatory changes. Wild-type mice received i.t. administration of PBS or LPS and were euthanized 24 hours later. Cells from BAL and lung tissue were isolated and sorted by flow cytometry, and *P2ry_6_* mRNA expression was assessed by qRT-PCR in **(A)** CD45^+^ hematopoietic cells, **(B)** CD11c-expressing cells, **(C)** Ly6G^+^ neutrophils, **(D)** F4/80^+^ macrophages, **(E)** CD4^+^ T cells, and **(F)** EpCAM^+^ epithelial cells. *P2ry_6_* mRNA expression was based on 2^-ΔΔCt^ values normalized to *Gapdh*. LPS challenge significantly increased *P2ry_6_* expression in immune cell populations but not in EpCAM^+^ epithelial cells. Data are presented as mean ± SEM; with the number of animals indicated in each graph. Statistical analysis was performed using Welch’s t-test; *P* < 0.05. **(G–I)** Representative H&E-stained lung sections showing inflammatory changes following LPS challenge. **(G)** Focal thickening of alveolar walls with intramural neutrophil accumulation (red arrows) and activated alveolar epithelial cells (black arrow). **(H)** Neutrophil accumulation within alveolar spaces and alveolar walls (red arrows). **(I)** Neutrophil accumulation in the alveolar space (black arrow), proteinaceous debris deposition (red arrows) and focal hyaline membrane formation (grey arrow). **(J–L)** Representative SFOG-stained lung sections demonstrating vascular alterations following LPS challenge. **(J)** Vascular congestion in small vessels and pulmonary capillaries (white asterisk and green arrows), focal intravascular coagulation (red arrow), and erythrocyte extravasation into the alveolar space (black arrow). **(K)** Hemorrhage in the peribronchiolar and perivascular regions (black arrows) and bronchial lumen (red arrow). **(L)** Congestion of small interstitial blood vessels (white arrow) and early thrombus formation (red arrows). Scale bar, 100 µm.

To characterize the inflammatory phenotype, lung sections were analyzed by H&E and SFOG staining. H&E staining revealed marked thickening of the alveolar septa, inflammatory cell infiltration and increased cellular accumulation within the alveolar and interstitial compartments (**Figures 3G–I**). SFOG staining demonstrated vascular congestion, focal hemorrhage and early thrombus formation within pulmonary capillaries (**Figures 3J–L**). Collectively, these findings support a potential cell type-specific role for *P2ry_6_* in the pathogenesis of LPS-induced ARDS. To better characterize the neutrophil structures, representative neutrophil-rich areas are shown at higher magnification (80×) which are corresponding to **Figures 3G–I** (**Supplementary Figure 3**).

### 3.2 Pharmacological inhibition of P2RY_6_ attenuates LPS-induced ARDS in prophylactic and therapeutic settings

To investigate the contribution of P2RY_6_ signaling to LPS-induced ARDS, we evaluated the effects of pharmacological P2RY_6_ inhibition using the selective P2Y_6_ receptor antagonist MRS2578 in prophylactic and therapeutic treatment settings. In the prophylactic regimen, mice (n= 5) received i.t. administration of MRS2578 prior to LPS challenge (**Figure 1A**). P2RY_6_ inhibition significantly reduced the accumulation of Ly6G^+^F4/80⁻ neutrophils and F4/80^+^Ly6G⁻ macrophages in the BAL (**Figures 1B, C**). This reduction was accompanied by decreased levels of pro-inflammatory cytokines and chemokines, including IL-1β (interleukin-1β), IL-6, TNF-α (tumor necrosis factor-α), MIP-2 (macrophage inflammatory protein-2), as well as partial reduction of KC (keratinocyte-derived chemokine) (*P* = 0.095) (**Figures 1D–H**). Isotype controls were applied to establish the gating strategy (**Supplementary Figure 1**). Furthermore, Evans blue extravasation analysis revealed that P2RY_6_ inhibition significantly reduced LPS-induced pulmonary microvascular leakage (**Figure 1I**). Histological examination of lung sections showed that prophylactic MRS2578 treatment substantially reduced LPS-induced ARDS compared with PBS-pretreated, LPS-challenged controls. In the PBS-pretreated LPS group, lung sections showed focal alveolar wall thickening, activation of alveolar epithelial cells and prominent neutrophil accumulation within alveolar walls and airspaces (**Supplementary Figures 4A–C**). In contrast, MRS2578-pretreated mice displayed largely preserved alveolar architecture, thin alveolar walls, only occasional alveolar macrophages and substantially reduced neutrophil accumulation in the alveolar compartment. Histological analysis of lung sections revealed attenuated lung injury in prophylactically MRS2578-treated mice compared with LPS control mice, characterized by reduced inflammatory cell infiltration and lung tissue damage (**Supplementary Figures 4D–F**).

To assess the therapeutic potential of P2RY_6_ inhibition, MRS2578 was administered after LPS challenge (n= 5) (**Figure 2A**). Similar to the prophylactic treatment, therapeutic inhibition of P2RY_6_ significantly reduced Ly6G^+^F4/80⁻ neutrophil and F4/80^+^Ly6G⁻ macrophage accumulation (**Figures 2B, C**), decreased pro-inflammatory cytokine and chemokine production, including IL-1β, IL-6, TNF-α and MIP-2, as well as partially reduced KC levels (**Figures 2D–H**). Consistently, Evans blue extravasation was significantly decreased following therapeutic P2RY_6_ inhibition (**Figure 2I**). Histological examination showed that therapeutic MRS2578 administration improved LPS-induced ARDS compared with LPS-challenged PBS-treated controls. PBS-treated mice showed neutrophil accumulation, alveolar-wall thickening, hyaline membranes and intra-alveolar proteinaceous debris (**Supplementary Figures 5A–C**), whereas MRS2578-treated mice showed preserved alveolar architecture and thin alveolar walls (**Supplementary Figures 5D–F**). Collectively, these findings demonstrate that pharmacological inhibition of P2RY_6_ attenuates inflammatory cell recruitment, cytokine production and vascular leakage in LPS-induced ARDS in both prophylactic and therapeutic settings.

To confirm the effective inhibition of P2RY_6_ by MRS2578, we performed a Ca^2+^ Flux assay as a functional readout of UDP-induced P2RY_6_ signaling. UDP alone induced a mean increase of 40.88%, whereas UDP in the presence of MRS2578 induced 13.33%, corresponding to approximately 67% inhibition of the UDP-evoked Ca²^+^ response by P2RY_6_ blockade (**Supplementary Figures 6A–C**). These results indicate that pharmacologic inhibition with MRS2578 effectively attenuates P2RY_6_-mediated Ca²^+^ signaling in BAL cells isolated from ARDS mice. Overall, the marked reduction in UDP-induced Ca²^+^ flux following MRS2578 treatment supports the functional attenuation of P2Y_6_-mediated responses in BAL cells from ARDS mice.

### 3.3 P2RY_6_ inhibition attenuates time-dependent PMN accumulation in the lung interstitium and BAL during LPS-induced ARDS

To further investigate the role of P2RY_6_ in regulating the spatial and temporal dynamics of neutrophil recruitment during ARDS, we quantified PMN migration in distinct pulmonary compartments, including the interstitium and alveolar space. Following LPS challenge, PMN numbers progressively increased in both the BAL and lung interstitium over a 48-hour period. PMN accumulation was consistently higher in the interstitium compared with the alveolar space, although both compartments displayed similar temporal kinetics (**Figures 4A, B**).

**Figure 4:**
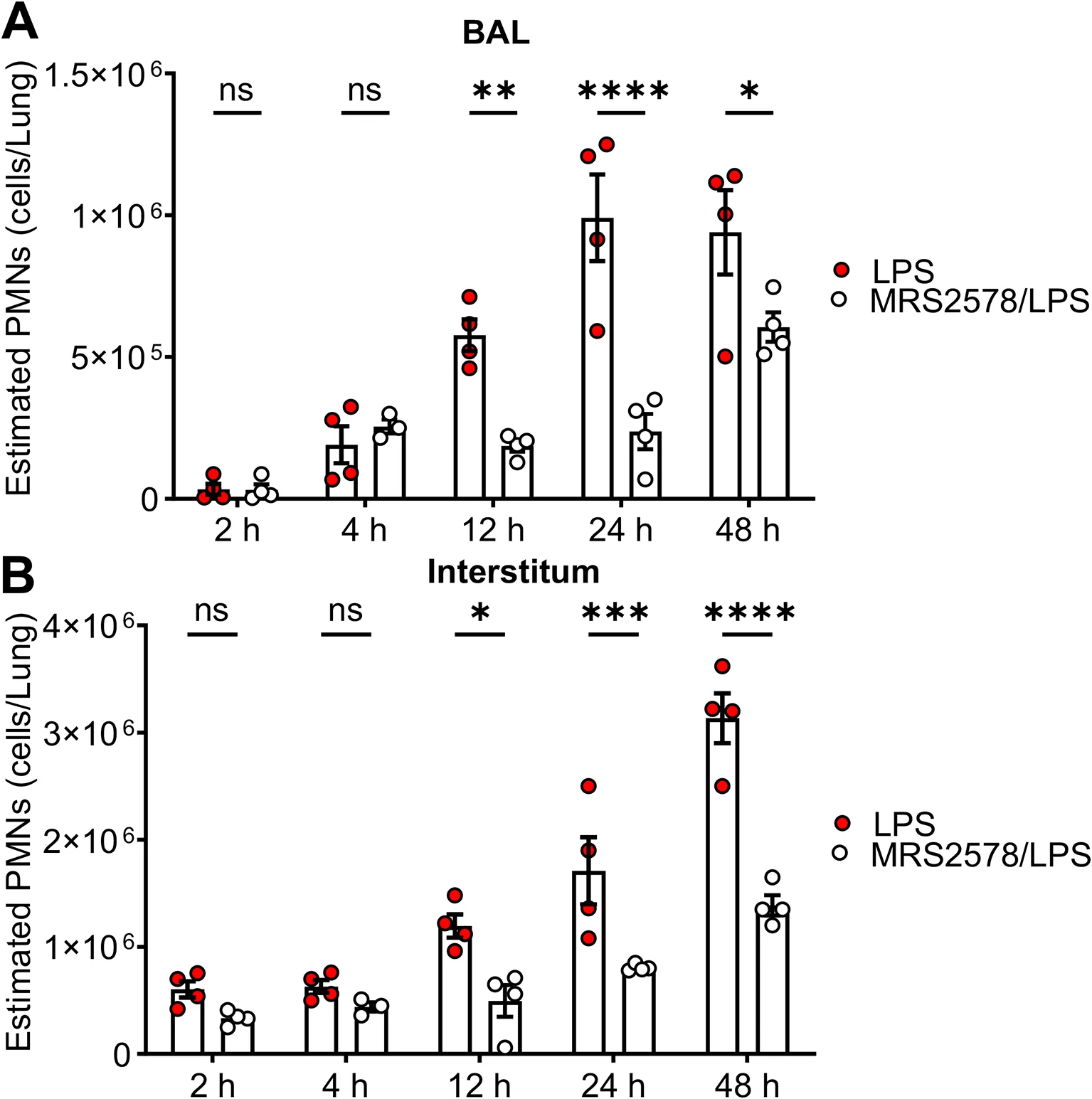
P2RY_6_ inhibition reduces time-dependent PMN accumulation in the alveolar and interstitial compartments during LPS-induced ARDS. PMN accumulation in distinct lung compartments was assessed following i.t. administration of Alexa 633-labeled rat anti-mouse GR-1 antibody (10 µg) in PBS/LPS- or MRS2578/LPS-treated mice over a 48 hours period. The estimated numbers of cells in BAL fluid and lung tissue per mouse were determined, and the frequency of PMNs within the different lung compartments was identified by flow cytometry. PMN accumulation in the **(A)** alveolar space and **(B)** lung interstitium was quantified by flow cytometry. Data are presented as mean ± SEM; the number of animals is indicated in each graph. Statistical analysis was performed using two-way ANOVA followed by Bonferroni’s multiple-comparisons test. *P < 0.05, **P < 0.01, ***P < 0.001, ****P < 0.0001. ns, not significant.

To determine the effect of P2RY_6_ inhibition on PMN recruitment, mice were treated with the selective P2Y_6_ receptor inhibitor MRS2578 prior to LPS challenge, and PMN accumulation was assessed over time. MRS2578 treatment significantly reduced PMN accumulation in both the BAL and lung interstitium from 12 to 48 hours after LPS challenge compared with LPS-treated controls (**Figures 4A, B**). Although early PMN recruitment during the first 4 hours after LPS instillation was not completely prevented, P2RY_6_ inhibition markedly limited subsequent PMN accumulation, with PMN numbers reaching a plateau at 12 hours and remaining stable through 24 hours. These findings demonstrate that P2RY_6_ contributes to the sustained recruitment and accumulation of PMNs during LPS-induced ARDS.

### 3.4 *P2ry_6_* deficiency attenuates inflammatory responses and lung injury in LPS-induced ARDS

To further investigate the contribution of P2RY_6_ to ARDS pathogenesis, we evaluated disease progression in global *P2ry_6_*-deficient mice. WT and *P2ry_6_^⁻/⁻^* mice were challenged with LPS for 24 hours, followed by analysis of BAL inflammatory cell populations and cytokine production. Compared with WT mice, *P2ry_6_^⁻/⁻^* mice exhibited a significant reduction in Ly6G^+^F4/80⁻ neutrophil accumulation in BAL following LPS challenge, whereas the number of F4/80^+^Ly6G⁻ macrophages was not significantly altered (**Figures 5A, B**).

**Figure 5:**
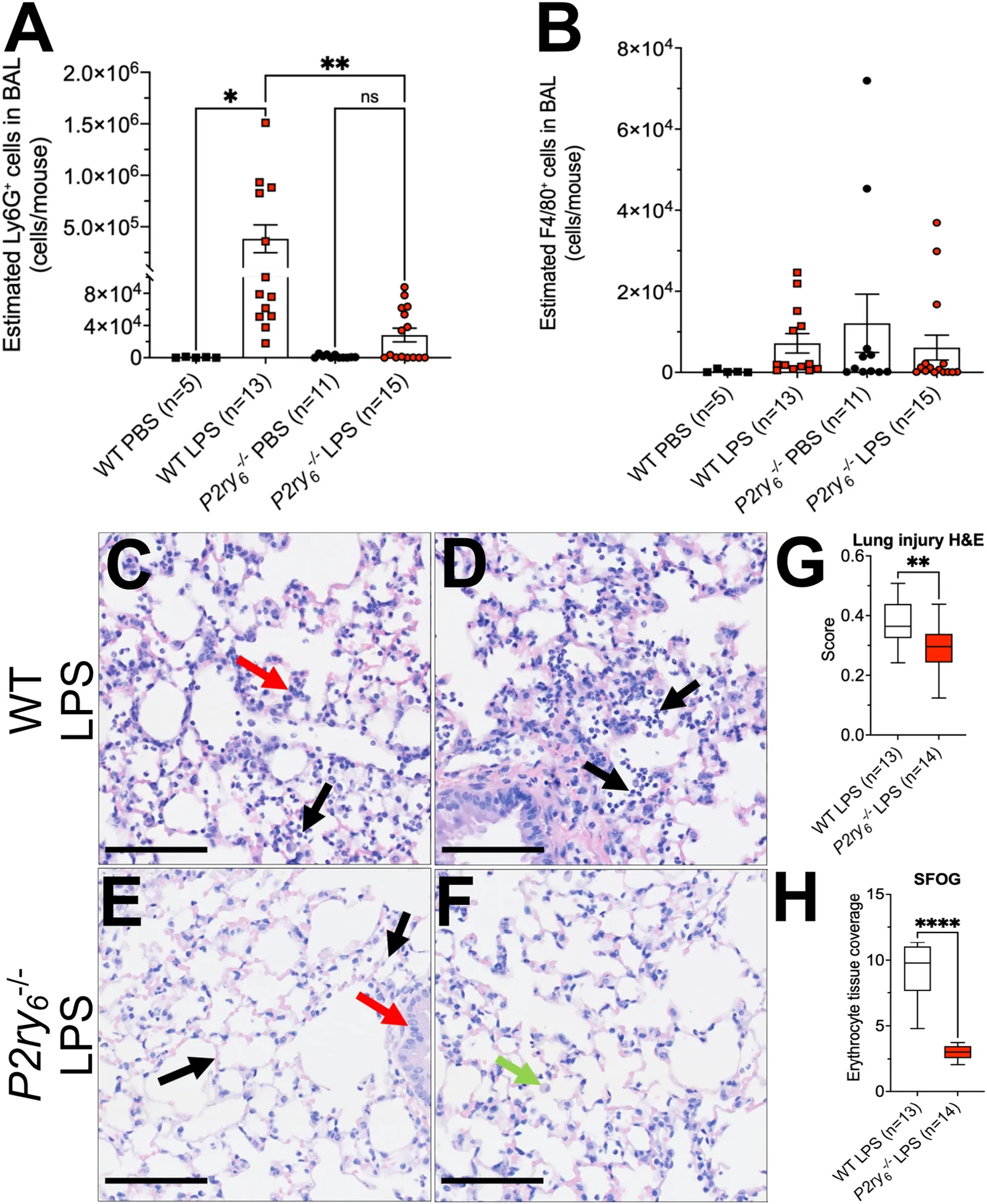
*P2ry_6_* deficiency attenuates inflammatory responses and lung injury in LPS-induced ARDS. Female *P2ry_6_^⁻/⁻^* and wild-type mice received i.t. administration of LPS or PBS. BAL fluid was collected and the estimated numbers of **(A)** Ly6G^+^F4/80⁻ neutrophils and **(B)** F4/80^+^Ly6G⁻ macrophages were quantified by flow cytometry and expressed as cells per mouse. Data are presented as mean ± SEM; the number of animals is indicated in each graph. Statistical analysis was performed using ordinary one-way ANOVA followed by Tukey’s multiple-comparisons test. *P < 0.05, **P < 0.01. **(C–F)** Representative H&E-stained lung sections from LPS-treated wild-type and *P2ry_6_^⁻/⁻^* female mice. **(C, D)** LPS-treated wild-type mice show focal alveolar wall thickening associated with activated alveolar epithelial cells and neutrophil accumulation (red arrows), with neutrophils also present within the alveolar spaces (black arrows). **(E, F)** LPS-treated *P2ry_6_^⁻/⁻^* mice show preserved lung architecture with thin alveolar walls (black arrows). The red arrow in panel E indicates preserved bronchiolar epithelium, and the green arrow indicates an occasional alveolar macrophage. Scale bars, 100 µm. Quantification of **(G)** lung injury score based on H&E staining and **(H)** erythrocyte tissue coverage based on SFOG staining was performed using the HALO^®^ image analysis platform. Data are presented as mean ± SEM; the number of animals is indicated in each graph. Statistical analysis was performed using Welch’s t-test. **P < 0.01, ****P < 0.0001.

Histological analysis revealed reduced lung injury in LPS-challenged *P2ry_6_^⁻/⁻^* mice compared with WT mice, characterized by decreased inflammatory cell infiltration and tissue damage (**Figures 5C–F**). Quantification of lung injury and erythrocyte tissue coverage further confirmed the protective effect of P2RY_6_ deficiency (**Figures 5G, H**). In addition, levels of inflammatory cytokines and chemokines, including IL-6, TNF-α, MIP-2 and KC, were significantly reduced in LPS-challenged *P2ry_6_^⁻/⁻^* mice compared with WT controls (**Figures 6A–D**). BALF from PBS-treated *P2ry_6_^⁻/⁻^* and WT mice was subjected to Bio-Plex Pro Mouse Cytokine 23-plex analysis. Most analytes were outside the reliable quantitative range, and KC was the only mediator consistently interpretable. KC did not change between WT and *P2ry_6_^⁻/⁻^* mice under PBS treatment (**Supplementary Figure 7**). Collectively, these findings demonstrate that P2RY_6_ promotes neutrophil recruitment, inflammatory mediator production and lung injury during LPS-induced ARDS.

**Figure 6:**
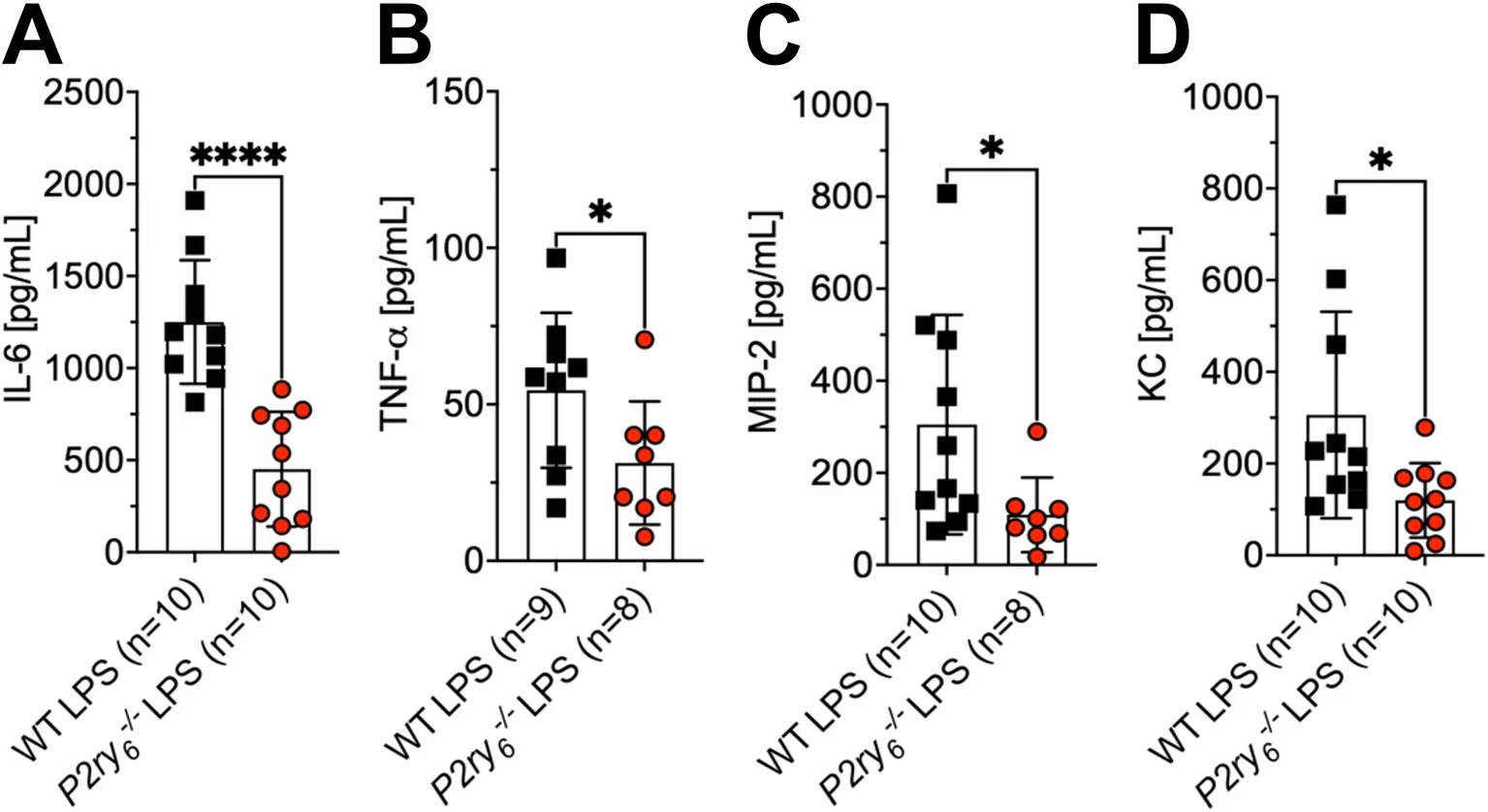
*P2ry_6_* deficiency reduces inflammatory cytokine and chemokine production in LPS-induced ARDS. BAL samples from LPS-treated *P2ry_6_^⁻/⁻^* and wild-type mice were analyzed using the Bio-Plex Pro Mouse Th17 Assays. Concentrations of pro-inflammatory cytokines **(A, B)** IL-6 and TNF-α and chemokines **(C, D)** MIP-2 and KC were measured in BAL supernatants from the indicated groups. Samples with cytokine or chemokine concentrations below the detection limit of the assay were excluded from the analysis. Data are presented as mean ± SEM; the number of animals is indicated in each graph. Statistical analysis was performed using Welch’s t-test. *P < 0.05, ****P < 0.0001.

### 3.5 Cell type-specific deletion of *P2ry_6_* in CD11c-expressing cells attenuates lung inflammation following LPS challenge

To further define the cell type-specific contribution of P2RY_6_ to ARDS pathogenesis, we generated conditional *P2ry_6_* knockout mice by crossing *P2ry_6_^fl/fl^* mice with different Cre-expressing lines targeting hematopoietic cells (*Vav*-Cre), CD11c-expressing cells (*CD11c*-Cre), myeloid cells (*LysM-Cre*), CD4^+^ T cells (*CD4*-Cre) and airway epithelial cells (*CCT*-Cre). Conditional knockout mice and corresponding controls were challenged i.t. with LPS for 24 hours, after which BAL and lung tissues were collected for analysis.

Flow cytometric analysis revealed a significant reduction in Ly6G^+^F4/80⁻ neutrophil accumulation in the BAL of *Vav*-Cre × *P2ry_6_^fl/fl^* and *CD11c*-Cre × *P2ry6^fl/fl^* mice following LPS challenge, whereas no significant reduction was observed in *LysM*-Cre × *P2ry_6_^fl/fl^*, *CD4*-Cre × *P2ry_6_^fl/fl^* or *CCT*-Cre × *P2ry_6_^fl/fl^* mice (**Figures 7A–E**). Consistently, histological analysis demonstrated reduced lung injury in LPS-challenged *Vav*-Cre × *P2ry_6_^fl/fl^* and *CD11c*-Cre × *P2ry_6_^fl/fl^* mice, as evidenced by reduced inflammatory changes in H&E-stained lung sections (**Figures 8A–H**). In contrast, BAL F4/80^+^Ly6G⁻ macrophage numbers were not significantly altered among the different conditional knockout groups (**Supplementary Figures 8A–E**). Among the measured inflammatory mediators (KC, IL-6, TNF-α and MIP-2) only KC was significantly reduced in LPS-treated *CD11c*-Cre × *P2ry_6_^fl/fl^* mice. This selective decrease in KC is consistent with the observed reduction in neutrophil accumulation in BALF and lung tissue, suggesting that attenuation of KC-dependent neutrophil recruitment is a major contributor to the protective phenotype. These data are provided as supporting evidence (**Supplementary Figure 9**). Collectively, these findings demonstrate that P2RY_6_ expressed by CD11c-expressing cells contributes to neutrophil recruitment and lung inflammation during LPS-induced ARDS in a cell type-specific manner.

**Figure 7:**
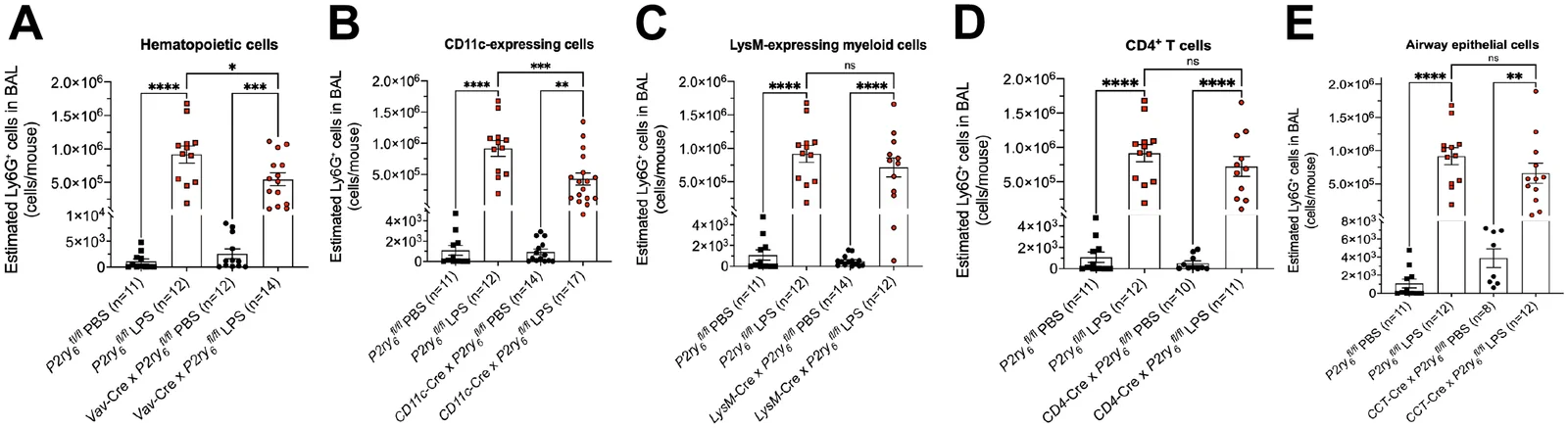
Deletion of *P2ry_6_* in CD11c-expressing cells attenuates inflammatory responses following LPS challenge. Conditional *P2ry_6_^fl/fl^* mice were crossed with *Vav*-Cre, *CD11c*-Cre, *LysM*-Cre, *CD4*-Cre, or *CCT*-Cre mice to generate cell-targeted *P2ry_6_* deletion models. Following LPS challenge, BAL fluid was collected, and the estimated number of Ly6G^+^F4/80⁻ neutrophils were quantified by flow cytometry and expressed as estimated number of cells per mouse in **(A)** *Vav*-Cre × *P2ry_6_^fl/fl^*, **(B)** *CD11c*-Cre × *P2ry_6_^fl/fl^*, **(C)** LysM-Cre × *P2ry_6_^fl/fl^*, **(D)** *CD4*-Cre × *P2ry_6_^fl/fl^*, and **(E)** *CCT*-Cre × *P2ry_6_^fl/fl^* mice. Data are presented as mean ± SEM; the number of animals is indicated in each graph. Statistical analysis was performed using ordinary one-way ANOVA followed by Tukey’s multiple-comparisons test. *P < 0.05, **P < 0.01, ***P < 0.001, ****P < 0.0001.

**Figure 8:**
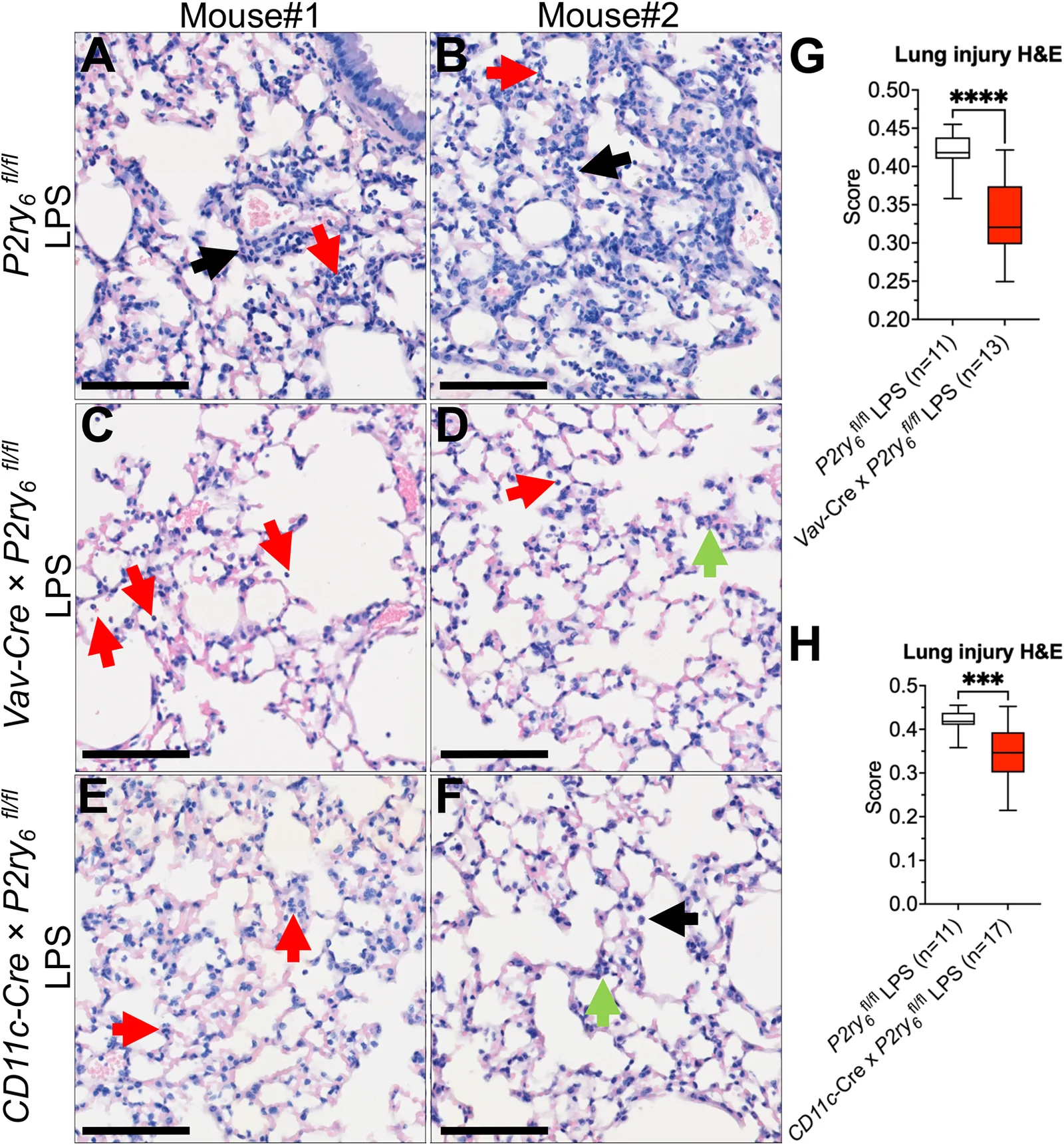
Histological assessment of lung injury in cell-targeted *P2ry_6_* deletion models following LPS challenge. Representative H&E-stained lung sections from LPS-treated control and conditional *P2ry_6_* deletion mice**. (A, B)** LPS-treated *P2ry_6_^fl/fl^* control mice exhibit marked inflammatory changes, including interstitial and alveolar inflammatory cell infiltration, focal alveolar wall thickening (black arrows), and neutrophil accumulation within alveolar spaces (red arrows). **(C, D)** LPS-treated *Vav*-Cre × *P2ry_6_^fl/fl^* mice show preserved lung architecture with thin alveolar walls. The alveolar spaces contain occasional alveolar macrophages and scattered neutrophils (red arrows), with limited interstitial granulocyte accumulation (green arrow). **(E, F)** LPS-treated *CD11c*-Cre × *P2ry_6_^fl/fl^* mice display preserved lung architecture with reduced inflammatory changes. The alveolar spaces contain few neutrophils (red arrows) and occasional alveolar macrophages (black arrow). Discrete cellular thickening of the alveolar wall is indicated by the green arrow. Scale bars, 100 µm. **(G, H)** Quantification of lung injury based on H&E staining using the HALO^®^ image analysis platform. Lung injury scores are shown for **(G)** *Vav*-Cre × *P2ry_6_^fl/fl^* LPS and **(H)** *CD11c*-Cre × *P2ry_6_^fl/fl^* LPS mice. Data are presented as mean ± SEM; the number of animals is indicated in each graph. Statistical analysis was performed using Welch’s t-test. ***P < 0.001, ****P < 0.0001.

## 4 Discussion

In the present study, we identified a critical cell type-specific role of P2RY_6_ in the progression of LPS-induced ARDS in female mice. This work was motivated by our previous findings demonstrating increased *P2ry_6_* mRNA expression in an LPS-induced ARDS model (26). Here, we further characterized the cellular distribution of *P2ry_6_* expression and observed increased expression in multiple BAL-derived populations, including CD45-positive cells, CD11c-expressing cells, Ly6G^+^ neutrophils, F4/80^+^ macrophages and CD4^+^ T cells in independent sorting experiments following LPS challenge. In contrast, *P2ry_6_* expression was not significantly increased in EpCAM^+^ epithelial cells. These findings suggest that P2RY_6_-mediated regulation of ARDS is primarily driven by immune cell populations rather than airway epithelial cells and indicate a potential hematopoietic and cell type-specific contribution to lung inflammation.

Our findings demonstrate that pharmacological inhibition of P2RY_6_ significantly attenuated key features of ARDS, including neutrophil accumulation, pulmonary vascular leakage and production of pro-inflammatory cytokines and chemokines in both prophylactic and therapeutic treatment settings. Although prophylactic and therapeutic administration of MRS2578 resulted in a partial reduction of KC levels, this may reflect the complex regulation of chemokine production during the inflammatory response, including contributions from multiple immune cell populations and signaling pathways (36). Furthermore, P2RY_6_ inhibition reduced PMN accumulation in the lung interstitium and BAL in a time-dependent manner, supporting a role for P2RY_6_ signaling in regulating sustained neutrophil recruitment during ARDS. Together, these findings suggest that targeting P2RY_6_ may represent a potential therapeutic strategy to limit neutrophil-driven lung injury.

Consistent with the pharmacological studies, global *P2ry_6_* deficiency significantly reduced Ly6G^+^F4/80⁻ neutrophil accumulation, inflammatory cytokine and chemokine production and histological features of lung injury following LPS challenge. These results extend previous studies demonstrating the involvement of purinergic signaling pathways, including P2X_7_ and P2Y_2_ receptors, in ARDS pathogenesis (12, 26).

To define the cellular compartments contributing to P2RY_6_-mediated inflammation, we employed conditional *P2ry_6_* knockout models targeting hematopoietic cells, CD11c-expressing cells, myeloid cells, CD4^+^ T cells and airway epithelial cells. Deletion of *P2ry_6_* in hematopoietic cells and CD11c-expressing cells significantly reduced neutrophil accumulation and lung injury following LPS exposure, whereas deletion in LysM^+^ myeloid cells, CD4^+^ T cells or airway epithelial cells did not reproduce this protective phenotype. Among the inflammatory mediators (KC, IL-6, TNF-α and MIP-2) measured in BALF after LPS challenge, only KC was significantly reduced in *CD11c*-Cre × *P2ry_6_^fl/fl^* mice compared with *P2ry_6_^fl/fl^* littermate controls. These findings support a mechanism in which P2RY_6_ signaling in CD11c-expressing cells amplifies neutrophil recruitment during LPS-induced lung inflammation by sustaining KC production. KC is a major neutrophil chemoattractant, and its reduction would be expected to weaken the chemotactic gradient that drives neutrophil influx into the lung. In this context, the reduced BALF and lung neutrophilia observed in *CD11c*-Cre × *P2ry_6_^fl/fl^* mice is consistent with impaired KC-dependent recruitment. Although we did not directly dissect the downstream signaling cascade in CD11c^+^ cells, our data indicate that P2RY_6_ contributes to ARDS pathology at least in part by promoting a chemokine program that supports neutrophil accumulation (37, 38). Previous studies have demonstrated that CD11c^+^ cells contribute to pulmonary inflammation and can regulate progression of acute lung injury (39, 40). Furthermore, the emergence of inflammatory cytokine-producing CD11c^+^ cells has been implicated in ARDS-like inflammatory conditions (41, 42). Consistent with these observations, our findings demonstrate that P2RY_6_ signaling in CD11c-expressing cells contributes to neutrophil recruitment and inflammatory responses during ARDS.

Notably, our previous work investigated a different purinergic receptor, P2RY_2_, and demonstrated that deletion of *P2ry_2_* in LysM-expressing cells using *LysM*-Cre × *P2ry_2_^fl/fl^* mice reduced LPS-induced ARDS inflammation (26). Together, these findings highlight that distinct purinergic receptors can regulate ARDS pathogenesis through different cellular compartments, with P2RY_2_-associated effects mediated through LysM-expressing cells and P2RY_6_-associated effects involving CD11c-expressing cells.

Although our conditional knockout approach provides important insights into the contribution of P2RY_6_ signaling within specific cellular compartments, several limitations should be acknowledged. The Cre drivers used in this study are not completely restricted to individual cell types, and their targeting patterns should therefore be interpreted with caution. *CD11c*-Cre mediates recombination in CD11c-expressing populations, including conventional dendritic cells and alveolar macrophages, while *LysM*-Cre targets LysM-expressing myeloid populations, including macrophages, monocytes and neutrophils. Thus, these models are more accurately described as CD11c- and LysM-expressing cell-targeted *P2ry_6_* deletion models rather than strictly cell type-specific knockouts. In addition, all experiments were performed exclusively in female mice. Given the well-established sex-dependent differences in immune responses and susceptibility to ARDS (43), the findings may not be directly generalizable across sexes. Future studies should determine whether P2RY_6_-expressing CD11c^+^ cells play a similar role in male mice or whether alternative immune cell populations contribute more prominently to ARDS pathogenesis in a sex-dependent manner. Complementary approaches, including inducible Cre systems, lineage-tracing strategies and cell-specific rescue models, will be valuable to further define the precise cellular contribution and temporal regulation of P2RY_6_ signaling during ARDS development.

In conclusion, this study provides the first direct *in vivo* evidence that P2RY_6_ contributes to LPS-induced ARDS through cell type-specific mechanisms in female mice. Our data demonstrate that P2RY_6_ promotes neutrophil recruitment, inflammatory mediator production and lung injury, with CD11c-expressing cells representing a key cellular compartment mediating these effects. These findings identify P2RY_6_ as a potential therapeutic target for controlling excessive inflammation and neutrophil-mediated tissue injury in ARDS.

## Data Availability

The original contributions presented in the study are included in the article/**Supplementary Material**. Further inquiries can be directed to the corresponding authors.
