# Supplementary material for: Cell-type-specific regulation of acute respiratory distress syndrome by P2RY6-expressing CD11c+ cells
Source: Front Immunol. 2026 Oct 26;17:1940896. doi: 10.3389/fimmu.2026.1940896 (PMC13745708; doi:10.3389/fimmu.2026.1940896)
Supplement: Supplementary file 2 [file DataSheet2.pdf]

frontiers

The International Society for the Advancement of Cytometry (ISAC) has highlighted the importance of including comprehensive methodological information to ensure data reproducibility and reliability. In line with this, Frontiers in Immunology now requires authors to submit a checklist for manuscripts that involve flow or mass cytometry. This checklist helps standardize the reporting process, improving the quality and transparency of published data. By doing so, we support scientific progress, making it easier for other researchers to replicate and validate experiments.

This form should be submitted with any manuscripts using flow or mass cytometry.

Sample/specimen/material description

☐ Total blood

☐ PBMCs

☐ Organ digests

Other Bronchoalveolar lavage cells/ Lung tissue

Did the samples suffer any treatment before or after incubation with the antibodies?

☐ Drug ___________________________________

☐ Cell permeabilization ______________________

☑ Dye Zombie yellow or DAPI

☐ Propidium iodine

☐ Not applicable

Other Mouse BD FcR Blocking reagent

Instrument and antibodies

Name of the Cytometer BD FACSAria I cell sorter

**Antibodies and targets**	**Fluorochrome/ Metal**	**Catalog number/Company**
~~e.g. anti-CD4~~	~~FITC~~	Cat. XXX/ XXX ltd.
anti-CD45	PerCP	Cat. 130-110-636/ Miltenyi Biotec

anti-CD11c	VioBlue	Cat. 130-122-939/
anti-CD4	FITC	Cat. 130-102-541/
anti-EpCAM	PE-Vio 770	Cat. 130-102-165/
anti-F4/80	PE-Vio 615	Cat. 130-123-913/
anti-Ly6G	FITC	Cat. 130-102-296/

Data analyses

1. Name of the software Aquired by BD FACSDiva 8.0.2, analyzed by Flowjo 10.10.1
2. Reference gating strategy in the manuscript or supplementary material

Gating strategy in (eg Figure X) Supplementary Figure 2
