# Supplementary material for: Cell-type-specific regulation of acute respiratory distress syndrome by P2RY6-expressing CD11c+ cells
Source: Front Immunol. 2026 Oct 26;17:1940896. doi: 10.3389/fimmu.2026.1940896 (PMC13745708; doi:10.3389/fimmu.2026.1940896)
Supplement: Supplementary file 3 [file SupplementaryFile1.docx]

**Supplementary Material**

This file contains additional methods and figures support the manuscript titled: Cell-Type-Specific Regulation of Acute Respiratory Distress Syndrome by P2RY_6_-Expressing CD11c⁺ Cells.

**Authors:** Zahra Kargarpour^1^, Bertram Aschenbrenner^2^, Markus Kramer^1^, Seda Metekol^1^, Amra Begunic^1^, Silvana Geleff^3^, Eva Obermayr^4^, Robert Zeillinger^4^, Anastasia Papaporfyriou^1^, Andreas Renner^1^, Beate Lichtenberger^2^, Daniela Gompelmann^1^, Marco Idzko^1*^, Ahmed El-Gazzar^1*^

**Supplementary Methods**

**Histology**

The P2RY_6_ selective inhibitor MRS2578 (TOCRIS Bioscience, UK, #2146) was administered i.t. (10 µM in 50 µL total volume) 30 minutes prior (prophylactic, n= 3) or 24 hours after (therapeutic, n= 3) i.t. LPS instillation. At the endpoint of LPS/inhibitor challenge, mice were euthanized and a BAL sample was obtained by cannulation of the trachea. Lungs were perfused by injecting of 3 mL PBS. The remaining lung tissue was slowly filled with histofix and transferred in formalin solution for histology analysis. Excised lungs from euthanized mice were perfused with 4% formaldehyde (Carl Roth GmbH, Karlsruhe, Germany) and processed into FFPE sections. Sections were deparaffinized in Neo-Clear, rehydrated and stained with filtered Mayer’s hematoxylin (Sigma-Aldrich; MHS-16). Following a double-distilled water rinse and 0.1% HCl differentiation, slides were eosin-counterstained, dehydrated in graded ethanol, cleared in butyl acetate, and mounted with Eukitt. All procedures and image analyses were performed by blinded investigators. Slides were scanned via a Vectra Polaris system (Akoya Biosciences, Marlborough, MA, USA) and manually evaluated utilizing HALO® software (v3.6.4134; Indica Labs, Albuquerque, NM, USA) (Supplementary Figures 4 and 5).

**Calcium Flux Assay**

Calcium flux was measured using the Calcium Flux Assay Kit (Abcam, Cambridge, UK, #ab233472) via flow cytometry, following the manufacturer's instructions. In brief, WT C57BL/6 mice (n=5) were i.t. instilled with LPS (1000 μg/kg body weight) to a total volume of 50 µL. After 24 hours, The BAL cells were collected, counted and 1.5 × 10^6^ cells were loaded with the calcium-sensitive dye by adding 1 µL of 520 AM dye stock solution to 0.5 mL of assay buffer solution per sample. The cells were then incubated for 30 minutes at 37°C. The cells were then treated with the P2RY_6_ inhibitor MRS2578 (10 µM) or left untreated. Subsequently, the cells were incubated for an additional 30 minutes at 37°C. During the final 10 minutes of this incubation period, 1 µg/mL of DAPI (Invitrogen, #D1306) was added to exclude dead cells. The cells were harvested by centrifugation at 330 g for 5 minutes and resuspended in 0.4 mL of HEPES-buffered saline solution (HBSS) containing 20 mM HEPES for subsequent flow cytometry analysis. A solution of 10 mM (5x) UDP in 25 µL or 10 mM ATP in 5 µL was prepared, yielding a final concentration of 500 µM or 100 µM in a total volume of 500 µl. Calcium flux was analyzed in the cells before and immediately after the addition of the prepared UDP solution (100 µL) using a CytoFLEX V5-B5-R3 flow cytometer at excitation/emission wavelengths of 490/525 nm (FITC). Changes in intracellular calcium were quantified by measuring mean fluorescence intensity (MFI) before and after stimulation. Acquisition was performed at a constant flow rate, with 120 seconds recorded per sample. Flow cytometry data were analyzed using FlowJo (version 10.10.1). MFI was measured prior to and following UDP stimulation, and the change in MFI (UDP-induced increase in MFI) was calculated by subtracting the baseline MFI from the post-stimulation MFI. Calcium flux was analyzed with dead cells excluded based on DAPI positivity. The UDP‑induced response was expressed as the percentage increase in MFI relative to the pre‑stimulation baseline within each experiment, calculated as follows: Percentage increase in MFI= $\frac{\text{MFI}_{\text{after UDP}}-\text{MFI}_{\text{before UDP}}}{\text{MFI}_{\text{before UDP}}}\times100$. ATP was included as a positive control to confirm assay responsiveness. The analysis were obtained from 4 independent experiments using total of 6 individual mice.

**Bio-Plex Multiplex Cytokine and Chemokine Assay**

WT and *P2ry_6_^⁻/⁻^* mice were treated with PBS, *CD11c*-Cre × *P2ry_6_^fl/fl^* mice and *P2ry_6_^fl/fl^* littermate control mice were challenged with LPS. BAL samples from PBS-treated *P2ry_6_^⁻/⁻^* (N= 7), wild-type (n= 5) mice, as well as LPS-challenged *CD11c*-Cre × *P2ry_6_^fl/fl^* (n= 12) and *P2ry_6_^fl/fl^* (n= 11) mice were subjected to a Bio-Plex Pro Mouse Cytokine 23-plex analysis (Bio-Rad Laboratories, #M60009RDPD) according to the manufacturer’s instructions (Supplementary Figures 7 and 9). This assay is a multiplex bead-based system that works similarly to a sandwich enzyme-linked immunosorbent assay (ELISA), allowing multiple analytes to be measured at the same time. The assay uses color-coded beads, each specific to a different analyte. These beads are first identified by a red laser, and the amount of bound analyte is then detected by measuring the fluorescence of a reporter molecule (phycoerythrin, PE) using a green laser. BAL samples and standards were prepared in 1xPBS containing 0.5% BSA to reduce nonspecific binding. All samples and standards were measured in duplicate. Standard curves were generated using a five-parameter logistic (5-PL) model and analyte concentrations were calculated based on the median fluorescence signal from at least 50 beads per analyte, using an eight-point standard curve. All measurements were performed using the Bio-Plex 200 instrument (Bio-Rad Laboratories). Analytes outside the reliable quantitative range were excluded.

**Supplementary figure legends**

**Supplementary Figure 1. Flow cytometry gating strategy for the identification of Ly6G⁺ neutrophils and F4/80⁺ macrophages in bronchoalveolar lavage fluid (BALF) from LPS-treated mice.** Wild-type mice were challenged with LPS and BALF was collected 24 hours later. Cells recovered from the BALF were analyzed by multicolor flow cytometry. BAL cells were initially gated based on forward scatter (FSC) and side scatter (SSC) properties, followed by doublet exclusion. Live single cells were subsequently analyzed for Ly6G and F4/80 expression. Positive gates were established using the corresponding isotype controls. **(A)** Representative staining of BAL cells from an LPS-treated mouse. **(B)** Representative isotype control staining. Numbers in each quadrant indicate the percentage of cells within the corresponding population.

**Supplementary Figure 2. Flow cytometry gating strategy for sorting of CD45^+^, CD11c^+^, CD4^+^, Ly6G⁺and F4/80⁺ cells from BAL fluid and EpCAM+ cells from lung tissue cells.** Wild-type mice were challenged with LPS and BAL fluid or lung tissue was collected 24 hours later. Cells recovered from the BAL fluid or lung tissue were analyzed by multicolor flow cytometry. Cells were initially gated based on FSC and SSC properties, followed by doublet exclusion. Live single cells were subsequently analyzed. **(A)** CD45^+^ cells were identified and sorted by gating on CD45-PerCPA against SSC-A. Subsequently, CD11c^+^ cells and CD4^+^ cells were sorted from CD45^+^ cell population by gating CD4-FITC against CD11c-VioBlue. **(B)** Ly6G^+^ and F4/80^+^ cells were sorted by gating Ly6G-FITC against F4/80-PEVio615. **(C)** EpCAM^+^ live cells isolated from lung tissue were sorted by gating EpCAm-PEVio770 against APC-A. Numbers in each quadrant indicate the percentage of cells within the corresponding population. For identification of live/dead cells in (A) Zombie yellow was used and in (B and C) DAPI was applied.

**Supplementary Figure 3. Characteristic histopathological features of lung injury following LPS challenge in mice.** Representative H&E-stained FFPE lung sections illustrating characteristic patterns of lung injury following LPS challenge. **(A)** Focal thickening of the alveolar walls due to activated alveolar epithelial cells, characterized by enlarged, round nuclei (inset, black arrow). Neutrophils, identified by their characteristic lobulated nuclei, also contribute to alveolar wall thickening (inset, upper red arrow). **(B)** Neutrophils with characteristic lobulated nuclei (red arrows) are observed both within the alveolar spaces (inset, upper left) and within the alveolar walls (insets, middle right and lower left). **(C)** Neutrophils with lobulated nuclei (red arrow) are present within the alveolar space (inset, upper left), while black arrows indicate deposits of proteinaceous debris. **Insets**: higher-magnification views acquired at 80×.

**Supplementary Figure 4. Characteristic histopathological features of lung injury following prophylactic inhibitor treatment.** The P2RY_6_ inhibitor MRS2578 attenuates LPS-induced ARDS. **(A–F)** Representative H&E-stained lung sections from C57BL/6 wild-type mice treated intratracheally (i.t.) with MRS2578 or PBS 30 min before i.t. LPS administration, using a prophylactic treatment protocol. Mice were euthanized 24 h after LPS challenge. **(A–C)** LPS-treated mice showed neutrophil accumulation within alveolar walls and airspaces (red arrows), together with focal alveolar wall thickening and activated alveolar epithelial cells (black arrows). **(D–F)** MRS2578-pretreated, LPS-challenged mice showed preserved alveolar architecture and thin alveolar walls (black arrows). Green arrows in **(E,F)** indicate occasional alveolar macrophages, and the red arrow in **(F)** indicates a granulocyte within a capillary. Scale bars, 50 μm.

**Supplementary Figure 5. Characteristic histopathological features of lung injury following therapeutic inhibitor treatment.** Therapeutic administration of the P2RY_6_ inhibitor MRS2578 attenuates LPS-induced ARDS. **(A–F)** Representative H&E-stained lung sections from female wild-type mice subjected to a therapeutic treatment protocol. Mice received intratracheal (i.t.) LPS administration, followed 24 h later by i.t. administration of MRS2578 or PBS. Mice were euthanized 24 h after MRS2578 or PBS administration (i.e., 48 h after LPS challenge). **(A–C)** LPS-challenged mice treated with PBS showed neutrophil accumulation within alveolar walls and airspaces (red arrows), focal alveolar-wall thickening with activated alveolar epithelial cells (black arrows; A), focal hyaline membrane formation (gray arrows; B), and proteinaceous debris within the alveolar space (orange arrow; C). **(D–F)** LPS-challenged mice treated with MRS2578 showed preserved alveolar architecture and thin alveolar walls (black arrows). The green arrow in **(D)** indicates an occasional alveolar macrophage. Scale bars, 50 μm.

**Supplementary Figure 6. UDP-induced Ca²⁺ responses in BAL cells from ARDS mice are attenuated by P2RY_6_ inhibition.** BAL cells were collected from C57BL/6 WT mice 24 h after intratracheal LPS administration (1,000 µg; n = 6 mice, pooled from four independent experiments). Cells were loaded with a Ca²⁺-sensitive fluorescent indicator and analyzed by flow cytometry. **(A)** Flow cytometry gating strategy for the identification of live cells in BAL fluid (BALF) from LPS-treated mice. BAL cells were initially gated based on FSC and SSC properties, followed by doublet exclusion. **(B)** Representative histograms of intracellular Ca²⁺ flux in BAL cells stimulated with UDP (500 µM, indicated in red) in the absence or presence of the P2RY_6_ antagonist MRS2578 (10 µM). ATP (100 µM, indicated in red) was used as a positive control to confirm cellular responsiveness and is shown as an additional histogram. Unstained cells (no Ca²⁺-sensitive fluorescent dye) are displayed in orange as a negative control. **(C)** Quantification of UDP-induced Ca²⁺ responses in BAL cells from WT C57BL/6 mice, with or without MRS2578. Ca²⁺ responses were expressed as the percentage increase in mean fluorescence intensity (MFI) relative to the pre-stimulation baseline within each experiment, using the formula:

$\text{UDP-induced increase in MFI (\%)}=\frac{\text{MFI}_{\text{after UDP}}-\text{MFI}_{\text{before UDP}}}{\text{MFI}_{\text{before UDP}}}\times100$.

Data are presented as mean ± SEM. Statistical significance was assessed using a two-tailed Welch’s t-test. ** *P* < 0.01.

**Supplementary Figure 7. Multiplex cytokine analysis of BAL fluid from PBS-treated WT and *P2ry_6_^⁻/⁻^* mice.** WT and *P2ry_6_^⁻/⁻^* mice were treated with PBS, and BAL fluid was collected 24 h later. BAL samples from PBS-treated *P2ry_6_*^⁻/⁻^ (N= 7) and wild-type (n= 5) mice were subjected to a Bio-Plex Pro Mouse Cytokine 23-plex analysis according to the manufacturer’s instructions. Concentrations of pro-inflammatory chemokine KC was shown in BAL supernatants from the indicated samples. Data are presented as mean ± SEM; the number of animals is indicated in each graph. Statistical analysis was performed using Welch’s t-test.

**Supplementary Figure 8. Cell type-specific deletion of *P2ry_6_* does not alter the number of F4/80⁺ macrophages in BAL fluid following LPS challenge.** Conditional *P2ry_6_^fl/fl^* mice were crossed with *Vav*-Cre, *CD11c*-Cre, *LysM*-Cre, *CD4*-Cre and *CCT*-Cre mice to generate cell type-specific *P2ry_6_* knockout mice. **(A-E)** Quantification of F4/80⁺ live cells recovered from BALF in *Vav*-Cre × *P2ry_6_^fl/fl^*, *CD11c*-Cre × *P2ry_6_^fl/fl^*, *LysM*-Cre × *P2ry_6_^fl/fl^*, *CD4*-Cre × *P2ry_6_^fl/fl^* and *CCT*-Cre × *P2ry_6_^fl/fl^* mice 24 hours following LPS challenge. Data are presented as mean ± SEM. n indicates the number of mice per group. Statistical analysis was performed using one-way ANOVA followed by Tukey’s multiple comparisons. * *P* < 0.05.

**Supplementary Figure 9. Selective reduction of KC in LPS-treated *CD11c*-Cre × *P2ry_6_^fl/fl^* mice.** *CD11c*-Cre × *P2ry_6_^fl/fl^* mice (n= 11) and *P2ry_6_^fl/fl^* (n= 12) littermate control mice were challenged with LPS, and inflammatory mediators were measured in BAL fluid 24 h later. Multiplex cytokine analysis was performed using the Bio-Rad Bio-Plex Pro Mouse Cytokine 23-plex assay according to the manufacturer’s instructions. Due to limited availability of BAL supernatant, all available *P2ry_6_^fl/fl^* samples were analyzed, whereas only 12 of 17 *CD11c*-Cre × *P2ry_6_^fl/fl^* samples could be included. The number of animals is indicated in the graph. Data are presented as mean ± SEM. Statistical analysis was performed using Welch’s t-test. * *P* < 0.05.
